# Integration of the Drug–Gene Interaction Database (DGIdb 4.0) with open crowdsource efforts

**DOI:** 10.1093/nar/gkaa1084

**Published:** 2020-11-25

**Authors:** Sharon L Freshour, Susanna Kiwala, Kelsy C Cotto, Adam C Coffman, Joshua F McMichael, Jonathan J Song, Malachi Griffith, Obi L Griffith, Alex H Wagner

**Affiliations:** Department of Medicine, Division of Oncology, Washington University School of Medicine, St Louis, MO 63110, USA; McDonnell Genome Institute, Washington University School of Medicine, St Louis, MO 63108, USA; McDonnell Genome Institute, Washington University School of Medicine, St Louis, MO 63108, USA; Department of Medicine, Division of Oncology, Washington University School of Medicine, St Louis, MO 63110, USA; McDonnell Genome Institute, Washington University School of Medicine, St Louis, MO 63108, USA; McDonnell Genome Institute, Washington University School of Medicine, St Louis, MO 63108, USA; McDonnell Genome Institute, Washington University School of Medicine, St Louis, MO 63108, USA; Department of Medicine, Division of Oncology, Washington University School of Medicine, St Louis, MO 63110, USA; McDonnell Genome Institute, Washington University School of Medicine, St Louis, MO 63108, USA; Department of Medicine, Division of Oncology, Washington University School of Medicine, St Louis, MO 63110, USA; McDonnell Genome Institute, Washington University School of Medicine, St Louis, MO 63108, USA; Department of Genetics, Washington University School of Medicine, St Louis, MO 63110, USA; Siteman Cancer Center, Washington University School of Medicine, St Louis, MO 63110, USA; Department of Medicine, Division of Oncology, Washington University School of Medicine, St Louis, MO 63110, USA; McDonnell Genome Institute, Washington University School of Medicine, St Louis, MO 63108, USA; Department of Genetics, Washington University School of Medicine, St Louis, MO 63110, USA; Siteman Cancer Center, Washington University School of Medicine, St Louis, MO 63110, USA; Department of Medicine, Division of Oncology, Washington University School of Medicine, St Louis, MO 63110, USA; McDonnell Genome Institute, Washington University School of Medicine, St Louis, MO 63108, USA; The Steve and Cindy Rasmussen Institute for Genomic Medicine, Nationwide Children's Hospital, Columbus, OH 43215, USA; Department of Pediatrics, The Ohio State University College of Medicine, Columbus, OH 43210, USA

## Abstract

The Drug-Gene Interaction Database (DGIdb, www.dgidb.org) is a web resource that provides information on drug-gene interactions and druggable genes from publications, databases, and other web-based sources. Drug, gene, and interaction data are normalized and merged into conceptual groups. The information contained in this resource is available to users through a straightforward search interface, an application programming interface (API), and TSV data downloads. DGIdb 4.0 is the latest major version release of this database. A primary focus of this update was integration with crowdsourced efforts, leveraging the Drug Target Commons for community-contributed interaction data, Wikidata to facilitate term normalization, and export to NDEx for drug-gene interaction network representations. Seven new sources have been added since the last major version release, bringing the total number of sources included to 41. Of the previously aggregated sources, 15 have been updated. DGIdb 4.0 also includes improvements to the process of drug normalization and grouping of imported sources. Other notable updates include the introduction of a more sophisticated Query Score for interaction search results, an updated Interaction Score, the inclusion of interaction directionality, and several additional improvements to search features, data releases, licensing documentation and the application framework.

## INTRODUCTION

Originally released in 2013, the Drug–Gene Interaction database (DGIdb) (1) serves as a central aggregator of information on drug-gene interactions and druggability from multiple diverse sources. The subsequent major updates to DGIdb 2.0 (2) (in 2016) and 3.0 (3) (in 2018) included improvements to the user interface and search response times, the addition of an API, the introduction and improvement of gene and drug grouping methods, and the expansion of source content through the inclusion of new sources and updates of existing sources. Since the release of DGIdb 3.0, many of the existing sources have been substantially updated and new sources have become available. Here we describe changes made for our most recent major version release, DGIdb 4.0. In this release, we have made an effort to integrate crowdsourced data and sources in several areas, including the addition of the crowdsourced Drug Target Commons (4) as a drug-gene interaction source, and the use of the open, community-curated Wikidata (5) resource for drug normalization. We also illustrate the value of our integration efforts in downstream community tools, through the incorporation of our data into NDEx (6). To keep content offered by DGIdb current, we have developed additional automatic update routines for multiple sources and implemented a new background job management system (Sidekiq, sidekiq.org) for routine job scheduling. Finally, DGIdb 4.0 focuses on numerous improvements of search results, including new and updated scores for interaction search results and improved drug normalization routines.

### Integration with crowdsourced efforts

**Figure 1.**
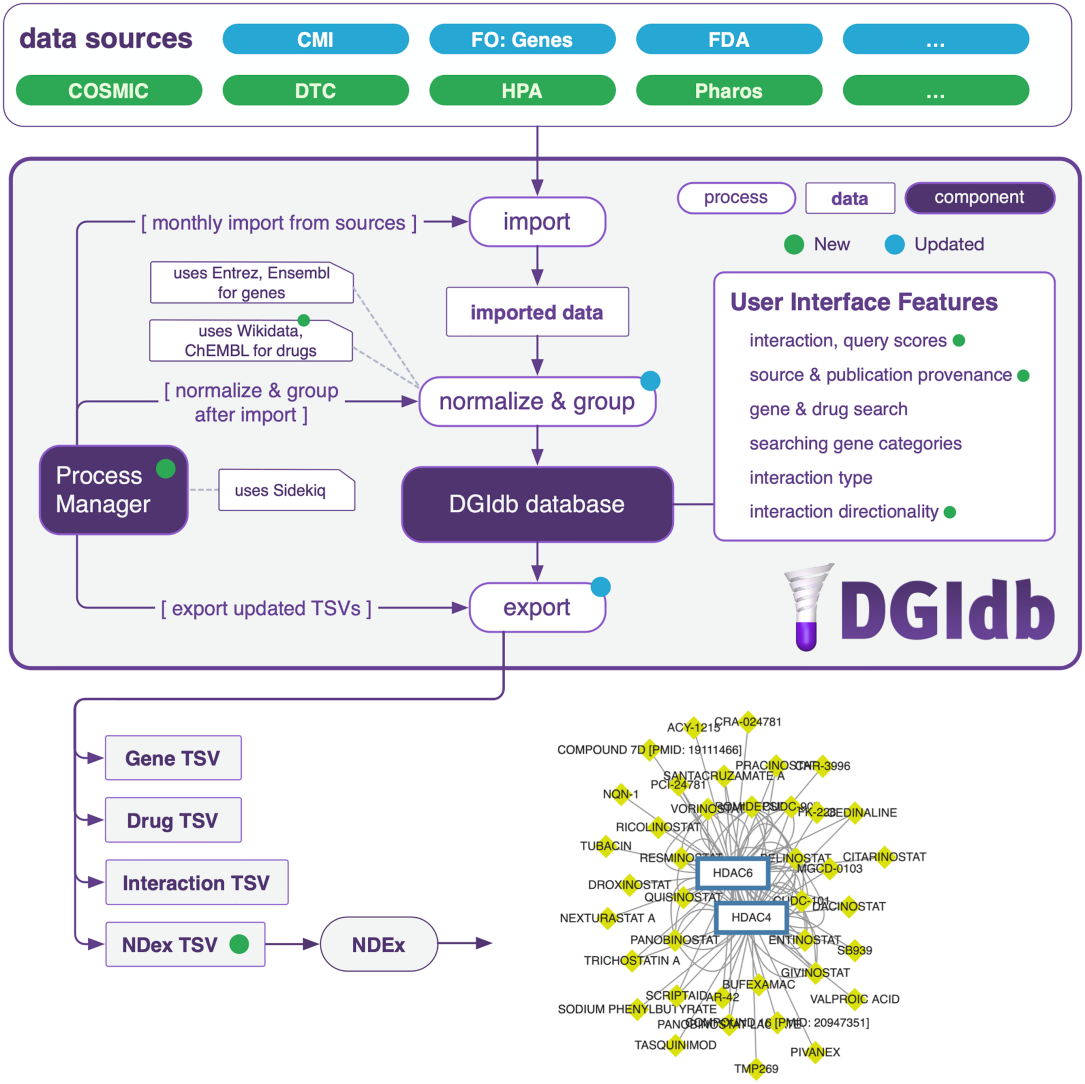
Overview of main components of DGIdb. Data sources are imported from outside resources (over 40 as of DGIdb 4.0), normalized and grouped with internal processes to prepare records to be displayed in DGIdb, and exported to TSV for download and integration with other resources. Process management is handled by Sidekiq for automation of importing, normalization and grouping, and exporting. A subset of new data sources are highlighted in green, a subset of updated pre-existing data sources are highlighted in blue. The updated sources highlighted in this figure are some of the sources that have been updated through manual curation. Information on additional sources and their status in DGIdb 4.0 can be found in Figure 2 and Supplementary Table S1. New features and technologies from DGIdb 4.0 are indicated with green dots, pre-existing features and technologies that have been updated are indicated with blue dots. The drug-gene network graph shown in the bottom right is an example of the data visualizations available on NDEx. Abbreviations: CMI = Caris Molecular Intelligence, FO = Foundation One, DTC = Drug Target Commons and HPA = Human Protein Atlas.

A primary focus of the DGIdb 4.0 release is the inclusion and utilization of crowdsourced efforts in several aspects of our database. The utility of our database begins with importing relevant drug, gene, and drug-gene interaction records (called *claims*) from outside resources. We normalize and sort these claims into conceptual *groups*, and make these concepts searchable via a web application and API. We also export data for bulk download and use with external resources (Figure 1). In this update, we extend these features by integration with crowdsourced drug-gene interaction claims, normalizing drug terms, and integrating with external resources.

For drug–gene interaction claims, we have added Drug Target Commons as a new source in DGIdb 4.0 (Figure 1). Drug Target Commons provides an extensive curated database of crowdsourced drug-gene interactions, from which we added a total of 23 879 interaction claims. This represents ∼24% (23 879/100 273) of the total interaction claims in DGIdb.

For drug normalization, we now use a Wikidata normalizer in addition to a ChEMBL (7) normalizer from the *thera-py* python package (Figure 1; additional detail in **Drug Grouping Improvements** section). Wikidata serves as a source of collaborative, crowdsourced drug concepts, and has allowed us to improve normalization in cases where ChEMBL normalization failed. For example, concepts representing the terms *annamycin*, *N-methyl scopolamine* and *Debio 1347* are all found in Wikidata but not ChEMBL.

Finally, we have integrated DGIdb with the Network Data Exchange (NDEx) (6), a community resource that allows sharing and publishing of biological data in a network-based format. For DGIdb, export of DGIdb data to the NDEx platform provides a resource for the visual representation of relationships and interactions between drugs and genes present in our database, allowing users to visually explore a global network of drugs and gene interactions of interest. NDEx TSVs are generated monthly and automatically uploaded to the NDEx server to keep the DGIdb network in NDEx up-to-date (Figure 1). NDex is the latest in a number of community resources that have integrated DGIdb. Existing data clients include GeneCards (8), BioGPS (9), CancerTracer (10), Gene4Denovo (11), SL-BioDP (12), TargetDB (13) and OncoGemini (14), among others.

### New and updated sources

**Figure 2.**
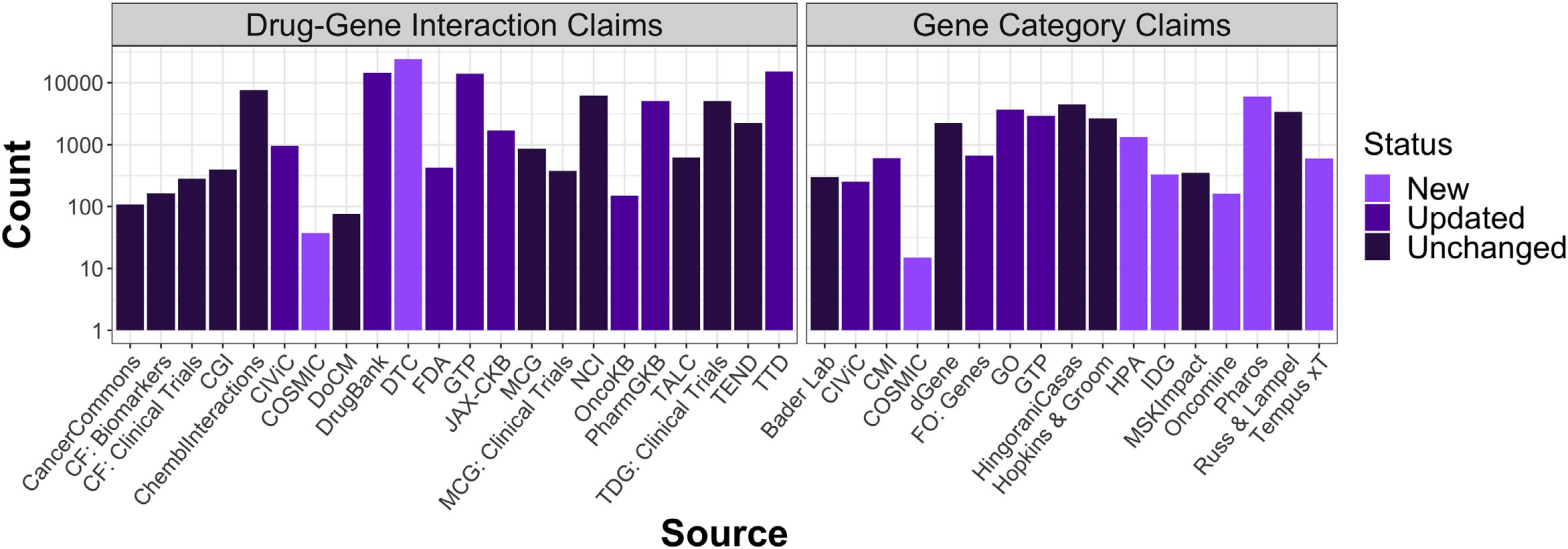
DGIdb 4.0 content by source. The number of drug-gene interaction claims (first panel) and druggable gene categories (second panel) are separated into three categories: sources that are new, sources that existed in the DGIdb previously but have been updated for 4.0, or sources that existed previously but have not been updated. Abbreviations: CF = Clearity Foundation, CGI = Cancer Genome Interpreter, CMI = Caris Molecular Intelligence, DTC = Drug Target Commons, FO = Foundation One, GO = Gene Ontology, GTP = Guide to Pharmacology, HPA = Human Protein Atlas, IDG = Illuminating the Druggable Genome, JAX-CKB = JAX-Clinical Knowledgebase, MCG = My Cancer Genome, MSK = Memorial Sloan Kettering, OncoKB = Precision Oncology Knowledge Base, TALC = Targeted Agents in Lung Cancer, TDG = The Druggable Genome, TEND = Trends in the Exploration of Novel Drug targets and TTD = Therapeutic Target Database.

In an effort to ensure that DGIdb offers diverse and contemporary information, we have updated and added several sources to DGIdb 4.0. In addition to the previously mentioned Drug Target Commons (4), we now also include COSMIC (15) as a new source of drug-gene interaction data (Supplementary Table S1). COSMIC also serves as an additional source of curated *Drug Resistance* gene category claims. Other new gene category sources include the Tempus xT (16) panel of actionable cancer therapy target genes, a list of the top priority genes from the Illuminating the Druggable Genome (IDG) (17) Initiative, the Human Protein Atlas (18), the Oncomine (19) clinical cancer biomarker assay, and understudied targets of the IDG program from Pharos (20) (Supplementary Table S1). From these new sources, we have added 23 916 new drug-gene interaction claims and 8478 new druggable gene category claims (Figure 2). In total, we have added two new sources of drug-gene interactions and five new sources of druggable gene category claims. DGIdb 4.0 now has 100 273 interaction claims and 33 577 druggable gene category claims. In total, there are now 10 606 druggable genes and 54 591 drug–gene interactions, which cover 41 102 genes and 14 449 drugs, within the DGIdb.

We have also updated multiple sources including large, well-curated sources such as DrugBank (21), Guide to Pharmacology (22), Gene Ontology (23,24), OncoKB (25), PharmGKB (26), and the Therapeutic Target Database (27) (Figure 2, Supplementary Table S1). To facilitate routine updates, the importer for PharmGKB has been updated to an online importer that can be run periodically using DGIdb's new automated job scheduling system (see Technology Improvements). Similarly, Pharos (20), one of our new sources, has been implemented as an online updater. Of the 41 sources in DGIdb, 12 sources are now imported using the online updater format, including Entrez (28), the core source of gene concepts for gene grouping in DGIdb, and Ensembl (29), a key source of gene aliases. In DGIdb 4.0, the number of genes imported from Entrez has increased from 41 102 to 42 851 and Ensembl has been updated from version 90_38 to version 101_38. We have also migrated several older sources from our original domain-specific language (DSL) importers to the improved TSV importer style implemented in DGIdb 3.0.

In DGIdb 4.0, the database structure and presentation model was updated to allow sources to be imported with multiple source types. This change enables merging of sources that were previously duplicated for each independent claim type (drug, gene, interaction, druggable gene category). For example, we previously imported both interaction claims and druggable gene category claims from Guide to Pharmacology (22) with two separate importers which created two separate sources (GuideToPharmacologyInteractions and GuideToPharmacologyGenes, respectively). With this update, the import of interaction claims and druggable gene category claims is now handled by one importer and only a single source (GuideToPharmacology) is created. This is intended to simplify and unify claim sources to aid in downstream interpretation. Additionally, supporting sources that have multiple source types enables easy extension to collect more informative claim type information. For example, some claims from CIViC (30) can be imported with the additional categories of drug resistance and clinically actionable. This change results in an additional 150 druggable gene category claims being imported from CIViC. Overall, these changes will increase the efficiency and accuracy of the process of importing and updating sources in DGIdb 4.0 compared to previous versions and will make it easier for users to evaluate individual sources.

### Drug grouping improvements

Another notable change in the DGIdb 4.0 update is the improvement to drug grouping and normalization. Previously, we grouped drug claims using a rule-based pairwise association approach. This process was cumbersome, requiring a lengthy and complete re-grouping of all claims whenever we updated sources in order to generate consistent groupings. We revised this approach by creating a normalization component independent of the claims aggregated by DGIdb, that could be run on a per-source basis. When redesigning this part of DGIdb, we took steps to enable reuse of this normalizer as a modular component for other resources. To this end, we leveraged and contributed to a drug normalization service from the Variant Interpretation for Cancer Consortium (the ‘*thera-py*’ Python Package; source code online at https://github.com/cancervariants/therapy-normalization). Among our contributions to *thera-py* was a normalizer for the Wikidata (5) resource, further enabling community contributions to assist in concept normalization both for DGIdb and other resources reliant upon the VICC normalization services.

Drug claims from DGIdb were normalized using the ChEMBL and Wikidata normalizers from *thera-py*. Rules were written to formalize grouper behavior based upon match characteristics of a query. Briefly, these rules prioritize matches to primary labels over aliases, exact case over case-insensitive, and ChEMBL over other normalizers. An algorithm for constructing a merged drug concept from normalizer results was specified, enabling a standardized set of aliases for a given concept identifier. Pseudocode for this algorithm is provided (see Supplementary Data), and all implemented code is available on our public repository (see Data Availability).

### New Query Score and updated Interaction Score

One of the main features added in DGIdb 4.0 is the concept of a relative *Query Score* for interaction search results. Previously, interaction search results displayed only a static *Interaction Score* based on evidence of an interaction (i.e. the number of publications and sources supporting an interaction claim). This Interaction Score did not take into account whether the gene and drug involved in a given interaction were also part of a large number of other interactions and, thus, had a low specificity that should be penalized. In addition, when searching for a set of genes or drugs, the Interaction Score does not prioritize results with overlapping interacting drugs or genes, which might be of more interest to the user, particularly in drug discovery and pathway applications.

**Figure 3.**
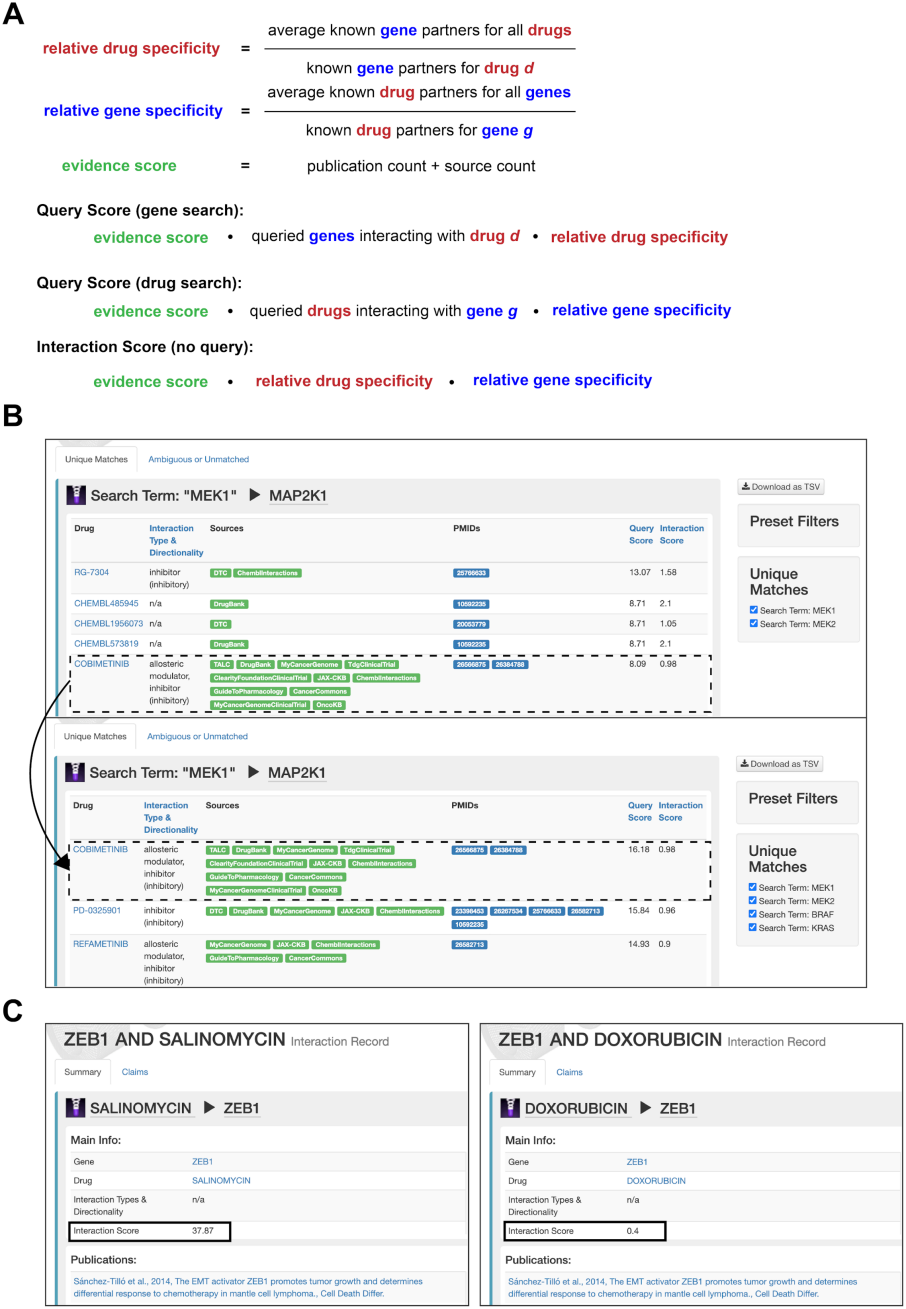
Overview of DGIdb's new Query scores and Interaction scores. (**A**) Schematic of how each of the new scores is calculated within DGIdb. Gene and drug queries both return a Query Score that is dependent on the search terms. Each interaction has an Interaction Score that is calculated independently of other search terms. (**B**) Example of a Query Score changing based on the terms searched. In the first panel, only *MEK1* and *MEK2* were searched and the Query Score for the interaction between *MEK1* and Cobimetinib was 8.09. In the second panel, *BRAF* and *KRAS* were added to the search query. These both interact with Cobimetinib and thus raise the Query Score to 16.18. (**C**) Example of Interaction Score. The panel on the left shows the interaction between *ZEB1* and Salinomycin. This is the only interaction for Salinomycin and thus it has a high Interaction Score. The panel on the right shows the interaction between *ZEB1* and Doxorubicin. Doxorubicin is involved in 103 interactions within DGIdb and thus has a much lower Interaction Score. Note that over time, as sources are updated and new claims are added, both Query Scores and Interaction Scores may change.

DGIdb 4.0 now provides a Query Score that is relative to the search set and considers the overlap of interactions in the result set. For interaction searches using a gene list, the Query Score is calculated from the Evidence Scores (publications and sources), the number of genes from the search set that interact with the given drug, and the degree to which the drug has known interactions with other genes (Figure 3). Similarly, for interaction searches using a drug list, the Query Score depends on the Evidence Scores (publications and sources), the number of drugs from the search set that interact with the given gene, and the degree to which the gene has known interactions with other drugs (Figure 3). In effect, this means that genes and drugs with many overlapping interactions in the search set will rank more highly, with the caveat that drugs or genes involved in many interactions, in general, will have lowered scores (Figure 3).

Our static Interaction Score previously introduced in DGIdb 3.0 (3) has been adjusted in DGIdb 4.0. The Interaction Score now mirrors the Query Score, except it is unaffected by the queried gene or drug sets, instead relying only on Evidence Scores and the degree to which both the gene and drug are involved in other interactions (Figure 3). Interaction Scores follow a long-tail distribution, indicative of many highly promiscuous (31) drugs and genes, and relatively few well-supported, highly specific drug-gene interactions (Supplementary Figure S1).

The introduction of the relative Query Score provides users with a score that gives a more intuitive ranking of drugs or genes based on the search set of interest, allowing the prioritization of drugs or genes that have overlapping, specific interactions with the search set. Similarly, the improvements to the static Interaction Score provide a more nuanced scoring system that takes into consideration the number of interactions for a drug-gene pair, in addition to the previous Evidence Scores, giving a more informative static Interaction Score.

As sources are updated and additional interaction claims are added to DGIdb, Interaction Scores and Query Scores are subject to change as a result of the changing measure to which Drug and Gene concepts interact with one another. Query Scores are always variable, dependent upon the set genes or drugs searched.

### Inclusion of interaction directionality

We have also added information on the directionality of interaction types to the interaction search results. Each interaction type in DGIdb now has an indicated directionality of *activating*, meaning the interaction type mechanism has an overall activating effect; *inhibitory*, meaning the interaction type mechanism has an overall inhibitory effect; or *n/a*, meaning the directionality is unclear for the interaction type mechanism (Supplementary Table S2). Determination of the directionality for an interaction type was made from mechanistic definitions provided by drug–gene interaction sources in which the interaction type was observed. Where these definitions were not available, we instead relied upon community definitions of these interaction types. These interaction directionalities are included on the UI for interaction search results in parentheses next to the interaction type(s) listed for each interaction result (Supplementary Figure S2). While the directionality may be obvious for some interaction types (e.g. activators are activating), some interaction types are not immediately apparent (e.g. chaperones are activating) to those less familiar with mechanisms of drug–gene interactions. Inclusion of directionality can make it easier for users to distinguish interactions that are more relevant for their purposes. For example, a user interested in exploring drugs that inhibit a particular gene will look for drug-gene interactions with inhibitory directionality. Users are also able to limit their interaction search to only interaction types of a desired directionality. Detailed information on each interaction type, the definition of each interaction type, and the directionality of each interaction are also available on the DGIdb Interaction Types page (https://dgidb.org/interaction_types).

### Search feature improvements

In DGIdb 4.0, we have introduced several updates related to searching, search results, and information available on the user interface. Among these are the addition of the option to search only cancer-specific sources (meaning sources that report claims relating to cancer only), or disease-agnostic sources (meaning sources that report claims relating to any disease, including cancer) for both interaction searches and druggable gene category searches. Cancer-related searches are a major use-case for DGIdb and cancer-specific sources are well-represented among all sources, with 13 cancer-specific drug-gene interaction sources and five cancer-specific druggable gene category sources. However, DGIdb is not a cancer-specific resource and is intended to be utilized for non-cancer related research as well. For drug-gene interactions, there are 4955 interactions supported by cancer-specific sources only, 48 341 interactions supported by disease-agnostic sources only, and 1,295 interactions are supported by both cancer-specific and disease-agnostic sources. Similarly, for druggable gene categories, 233 genes have categories supported by cancer-specific sources only, 17 168 genes have categories supported by disease-agnostic sources only and 2804 genes have categories supported by both cancer-specific and disease-agnostic sources. These numbers show that although a sizable portion of the sources included in DGIdb are cancer-specific, those types of sources only represent a small proportion of the overall data.

Other improvements to search result features include the addition of linkouts to specific interaction evidence, where available. These will allow users to browse to the primary source for an interaction claim which might provide additional information and context not captured by DGIdb. Also, while we introduced drug and gene filters to the interaction search view in the last major update, we have had several requests to define how these filters are implemented. To address this lack of transparency on the UI, we have now added a link to the FAQ page where these filters are now defined.

### Monthly data releases

DGIdb 3.0 implemented online updaters that imported data from dynamic sources (such as CIViC (30), Guide to Pharmacology (22), OncoKB (25), etc.) periodically, usually monthly. As a result, the static TSV data releases available on our Downloads page would quickly become outdated. For DGIdb 4.0, we have implemented monthly data releases of these TSVs to coincide with monthly runs of the online updaters, to ensure that TSVs available for download reflect the most up to date information in our database. The Downloads page now makes available the current Gene, Drug, Interaction and Category TSVs as well as previous monthly TSVs since the release of DGIdb 4.0. These serve as *de facto* snapshots of the data in DGIdb over time.

### Improved transparency and details on licensing of sources

In DGIdb 4.0, we have made a significant effort to update and improve the information we provide on licensing of sources imported into our database through manual curation of data license descriptions and references for every source. This information is now readily available on the sources page. Since DGIdb 3.0, several existing sources have made changes to their licensing, making data from some sources more broadly available and data from other sources more restricted. Notably, PharmGKB (26) has moved to a more permissive Creative Commons Attribution-ShareAlike 4.0 International License and DrugBank (21) has adopted a custom non-commercial license. In contrast, OncoKB (25) has restricted API access to registered/approved non-commercial research use only, and JAX-CKB (32) has restricted API access to negotiated licenses only. Both resources continue to provide access to a portion of their data for free through their respective web clients.

### Application framework updates

To handle increased web traffic and integration with other tools, we have upgraded DGIdb to Rails 6 (from Rails 5), upgraded to Ruby 2.6.5 (from Ruby 2.3), upgraded to PostgreSQL 12 (from PostgreSQL 9.6), and upgraded the server to the latest Ubuntu LTS release (20.04). In addition to the new features and performance benefits these upgrades bring, they will ensure that we continue to remain on supported software versions that receive regular security updates.

In order to keep DGIdb's underlying source data current, we had previously implemented an automated job scheduling framework using DelayedJob to schedule monthly runs of online updaters. In this release, we switched to using Sidekiq. In contrast to DelayedJob, Sidekiq offers a convenient user interface which makes identifying job failure reasons and rescheduling of failed jobs easier. Furthermore, the addition of Airbrake (https://airbrake.io/), an online tool for exception tracking, gives error reviews and notifies the development team of these errors in real-time (for instance, via email).

To ease future implementation of fixes and new features, we moved testing to a GitHub continuous integration (CI) workflow which allows us to continuously test newly committed code for errors against multiple versions of Ruby and PostgreSQL.

## SUMMARY AND FUTURE DIRECTIONS

With our most recent release, DGIdb has received significant improvements to source content, functionality such as searching and grouping, and underlying application technology. We have significantly expanded the number of records in our database through the addition of new sources and updates of existing sources. Furthermore, we have improved our ability to maintain regular content updates through the implementation of additional online importers for several sources and the use of Sidekiq for automatic job processing. We have revised our process for drug grouping and normalization to be batched by resource and to leverage continual improvement through community contributions to the VICC *thera-py* normalizers and the Wikidata public-domain crowdsourcing platform. Finally, several updates have been made to inform users of the relevance of search results through information presented on the UI. We have implemented more sophisticated notations of relative and static interaction scores, improved the relevance of interaction source linkouts wherever possible, and included the concept of directionality for interaction results.

Although the updates in DGIdb 4.0 have improved the usability and content of our resource, we expect there will still be a need for future improvements. One technology improvement on our roadmap is converging the public-facing API with the internal code that powers the web views. Ultimately, we want the APIs available for general use to be the same ones powering our HTML pages. This would provide an even more fully featured API to end users while reducing our overall maintenance burden by eliminating redundant code. We are also evaluating the addition of information on gene-gene relationships. As always, we plan to continue updating sources to online updaters where possible, and migrating TSV-based sources from the legacy DSL importers to the TSV importers introduced in DGIdb 3.0.

## DATA AVAILABILITY

DGIdb is an open access database and web interface (www.dgidb.org) with open source code available on GitHub (https://github.com/griffithlab/dgi-db) under the MIT license. We also provide data downloads for drug claims, gene claims, and interaction claims on the website in addition to a SQL data dump (http://dgidb.org/downloads). Information about the API and its endpoints can also be found on the website (http://dgidb.org/api).

## Supplementary Material

gkaa1084_Supplemental_File
