# Supplementary material for: Integration of the Drug–Gene Interaction Database (DGIdb 4.0) with open crowdsource efforts
Source: Nucleic Acids Res. 2020 Nov 25;49(D1):D1144–51. doi: 10.1093/nar/gkaa1084 (PMC7778926; doi:10.1093/nar/gkaa1084)
Supplement: gkaa1084_Supplemental_File [file gkaa1084_supplemental_file.pdf]

SUPPLEMENTARY DATA

TABLES:

Supplementary Table 1: Description of Sources in DGIdb 4.0

Data Type	Source	Description of Sources and Imported Data	4.0 Status	Cancer-Specific?
Druggable Gene Categories	Bader Lab(1)	A publication detailing four large protein families. These gene category claims are available from the supplementary data hosted online, which were downloaded for import into the DGIdb.	P, S	No
Drug Gene Interactions	Cancer Genome Interpreter (CGI) (https://www.cancergenomeinterpreter.org/)(2)	CGI is a database that provides information about identified alterations and currently available treatment treatment. The biomarkers per variant file was downloaded and parsed for import into the DGIdb.	A, P	Yes
Drug Gene Interactions	CancerCommons(3)	CancerCommons provides a number of drugs that are approved or undergoing clinical trials for use in lung, prostate, and skin cancer. Drug-gene interactions were extracted from their web pages describing these diseases and imported.	P	Yes
Druggable Gene Categories	Caris Molecular Intelligence(https://www.carismolecularintelligence.com/)	We extracted clinically actionable gene lists from the biomarker and NGS panels provided by Caris Life Sciences online at http://www.carismolecularintelligence.com/.	U	Yes
Drug Gene Interactions	ChEMBL: Interactions(4)	ChEMBL is a database of small molecules capable of bioactivity that have been annotated with metadata such as 2-D structures and calculated properties (e.g. Molecular Weight). Drug-gene interactions were pulled from the database.	P	No
Drug Definitions	ChEMBL:Drugs(4)*	ChEMBL is a database of small molecules capable of bioactivity that have been annotated with metadata such as 2-D structures and calculated properties (e.g. Molecular Weight). ChEMBL serves as a source of drug concepts for normalization.	A, U	No
Drug Gene Interactions, Druggable Gene Categories	CIViC(5)	CIViC is a community-driven platform for identifying actionable variants in cancer. CIViC genes for Predictive evidence items were pulled via the provided API. Drug-gene interactions and druggability of those genes were imported. All genes imported from CIVIiC were assigned the DRUG RESISTANCE gene category. In addition, genes with level A Predictive evidence items were assigned the CLINICALLY ACTIONABLE gene category.	A, U	Yes
Drug Gene Interactions	Clearity Foundation: Biomarkers	The Clearity Foundation has analyzed thousands of tumors from ovarian cancers and retrospectively identified biomarkers that predict treatment response to select drugs in patient ovarian tumors. These interaction data were extracted from their web page and imported.	P, S	Yes
Drug Gene Interactions	Clearity Foundation: Clinical Trials	124 curated clinical trial records were provided by The Clearity Foundation based on their relevance to breast and ovarian cancer. The interactions from these trials were imported.	P, S	Yes
Drug Gene Interactions, Druggable Gene	COSMIC(6)	COSMIC provides curated information on cancer mutations and drug resistance. A CSV file was manually created from the table available at https://cancer.sanger.ac.uk/cosmic/drug_resistance for import in DGIdb. All genes that were imported were assigned	N	Yes

Categories		to the DRUG RESISTANCE gene category.		
Druggable Gene Categories	dGENE(7)	An annotation tool for checking if genes are members of one of ten druggable gene families. The lists of these gene families were provided by the authors and imported into the DGIdb.	P, S	No
Drug Gene Interactions	DoCM(8)	DoCM is a manually curated database of mutations associated with cancer progression. Drug-gene interaction data were pulled via the API and imported into the DGIdb.	A, P	Yes
Drug Gene Interactions	Drug Target Commons(9)	DTC is an extensive database of drug-gene interactions that have been curated through crowd-sourcing curation of expert knowledge. This data was downloaded from the Drug Target Commons website as a raw text preprocessed to only keep entries that have a compound_name, gene_names, and have a value of "active" or "Active" in the activity_comment column before import into DGIdb	N	No
Drug Gene Interactions	DrugBank(10)	DrugBank is a large community resource detailing drug to drug target information. The full DrugBank database was downloaded as an xml file and parsed for import into the DGIdb.	A, U	No
Gene Definitions	Ensembl(11)	Ensembl gene IDs were imported and linked to Entrez gene records. These were imported from Ensembl's ftp site using the transcript GTF file, and also the NCBI gene_info file.	A, U	No
Drug Gene Interactions	FDA Biomarkers (https://www.fda.gov/drugs/science-and-research-drugs/table-pharmacogenomic-biomarkers-drug-labeling/)	The FDA provides drug-gene interactions in their Pharmacogenomic Biomarkers in the Drug Labeling table. 427 drug-gene interactions were pulled from this table for various diseases.	U	No
Druggable Gene Categories	Foundation One Genes(12)	Foundation One diagnostic tests focus on clinically actionable genes, which are available via an online table on their website. These data were extracted from the site for import into the DGIdb.	U	Yes
Druggable Gene Categories	GO(13, 14)	Multiple gene categories were expertly selected and an online updater was created which queries the Gene Ontology API for these categories for import into the DGIdb.	A, U	No
Drug Gene Interactions, Druggable Gene Categories	Guide To Pharmacology(15)	The Guide to Pharmacology is an extensive resource detailing pharmacological targets and their corresponding drugs. The online updater downloads the interaction and gene files and then parses them for import into the DGIdb. Gene categories were assigned based on the Type field in the gene file.	A, U	No
Druggable Gene Categories	HingoraniCasas(16)	A publication from the Casas lab that used computational approaches to identify druggable genes from genome-wide association studies. Interaction claims were curated from this paper's supplementary information.	P, S	No
Druggable Gene Categories	Hopkins & Groom(17)	Considered the 'original' druggable genome by the DGIdb 1.0, information on genes that were predicted to make good drug targets were extracted from the paper for import into the DGIdb.	P, S	No
Druggable Gene Categories	Human Protein Atlas(18)	Used a computer-based modeling approach to identify genes predictive of cancer outcome. A TSV was created by going to the https://www.proteinatlas.org/search/protein_class%3APotential+drug+targets page and selecting the columns Gene, Gene synonym, Ensembl gene id, Gene description, Uniprot accession, and Protein class (from the Gene section) and clicking "Download TSV". This TSV was then imported into DGIdb.	N	No

Druggable Gene Categories	Illuminating the Druggable Genome (IDG) Initiative(19)	IDG focuses on understudied targets in three broad classes of druggable genes: G protein-coupled receptors, ion channels, and kinases. A TSV file was created manually from the table available at https://druggablegenome.net/IDGProteinList for import into DGIdb.	N	No
Drug Gene Interactions	Jax-Clinical Knowledgebase (JAX-CKB) (20)	JAX-CKB is a knowledge base that provides drug-gene interactions based on a tumor's genomic profile as well as drug efficacy and resistance evidence. Drug-gene interactions and metadata were pulled via the API and imported into the DGIdb.	A, U	Yes
Druggable Gene Categories	MSK IMPACT(21)	Clinically actionable genes were extracted from the supplementary tables of the Memorial Sloan Kettering IMPACT paper.	P, S	Yes
Drug Gene Interactions	My Cancer Genome(22)	My Cancer Genome provides information on how mutations drive cancer, and the implication of those mutations for treatment, including linking interactions between specific mutations and therapies. These interactions were extracted from their website with permission.	P, S	Yes
Drug Gene Interactions	My Cancer Genome: Clinical Trials(22)	My Cancer Genome previously supported the searching of clinical trials to support their interactions. When this was available, these data were extracted from their website with permission and imported into the DGIdb.	P, S	Yes
Drug Gene Interactions	National Cancer Institute (NCI) Cancer Gene Index (https://wiki.nci.nih.gov/display/cageneindex)	This resource was curated through scraping interactions from publications followed by manual curation. The Gene-Compound xml file was downloaded and parsed for inclusion into the DGIdb.	P, S	Yes
Gene Definitions	NCBI Entrez Gene(23)	Entrez gene serves as the canonical concept for genes in the DGIdb. Entrez Gene records are imported from NCBI using the online gene_info file.	A, U	No
Drug Gene Interactions	OncoKB: a Precision Oncology Knowledgebase(24)	OncoKB is a knowledgebase that provides information about the specific effects of a somatic molecular alteration and potential treatment options with predicted drug response information. Drug-gene interactions and metadata were pulled via the API and imported into the DGIdb.	A, U	Yes
Druggable Gene Categories	Oncomine(https://www.oncomine.com/)(25)	A clinical cancer biomarker assay. A CSV was manually created from the table available in the Comprehensive Assay V3 flyer (https://assets.thermofisher.com/TFS-Assets/LSG/brochures/oncomine-comprehensive-assay-v3-flyer.pdf) for import into DGIdb.	N	Yes
Drug Gene Interactions	PharmGKB(26)	PharmGKB is a knowledge resource that collects information about potentially clinically actionable gene-drug associations. These associations were downloaded via the relationships.tsv file from PharmGKB for parsing and import into the DGIdb.	A, U	No
Druggable Gene Categories	Pharos(27)	Hosts data as part of the IDG program. An online updater was created, which queries the Pharos GraphQL API (https://pharos-api.ncats.io/graphql) for gene category data.	A, N	No
Drug Definitions	PubChem(28)	PubChem served as a canonical concept for drugs in the DGIdb 3.0. It has been removed in 4.0 as the updated drug grouper relies on normalized concepts from ChEMBL and Wikidata	R	No
Druggable Gene Categories	Russ & Lampel(29)	Considered the 'updated' druggable genome by the DGIdb 1.0, information on genes that were predicted to make good drug targets were sent by the authors for import into the DGIdb.	P, S	No

Drug Gene Interactions	Targeted Agents in Lung Cancer (TALC)(30)	This 2012 publication presented a comprehensive survey of targeted agents in lung cancers. The data were provided as pdf tables within the publication and were manually reviewed for import into the DGIdb.	P, S	Yes
Drug Gene Interactions	TDG Clinical Trials(31)	This 2014 publication evaluated drug-target interactions in the CenterWatch Drugs in Clinical Trials Database. The aggregated drug-gene interactions were provided as a supplementary table, which was manually reviewed for import into the DGIdb.	P, S	No
Druggable Gene Categories	Tempus xT(32)	Panel of actionable cancer therapy target genes. A TSV was manually created from the gene list published by Tempus (https://www.tempus.com/wp-content/uploads/2018/12/xT-Gene-List_112818.pdf) for import into DGIdb.	N	Yes
Drug Gene Interactions	Therapeutic Target Database (TTD)(33)	The periodically updated TTD resource provides information about therapeutic targets and their corresponding drugs. This information was downloaded from the TTD website as raw text, and then parsed for import into the DGIdb. Drugs that were listed as 'Terminated', 'Withdrawn from market', or 'Discontinued' were not included.	U	No
Drug Gene Interactions	Trends in the Exploitation of Novel Drug Targets (TEND)(34)	This 2011 publication was an extensive manual curation of FDA approved drugs and their targets from DrugBank. The results of this effort are in a supplementary table, which was manually reviewed for import into the DGIdb.	P, S	No
Drug Definitions	Wikidata(35)	Wikidata is a free and open knowledge base containing structured data that can be read and edited by both humans and machines. Wikidata serves as a source of drug concepts for normalization.	A, N	No

A: Denotes source that is automatically updated, N: Denotes source that is new in the DGIdb 4.0, P: Denotes source that was previously in the DGIdb 3.0 and has not been updated, R: Denotes a source that was previously in DGIdb 3.0 but has been removed in 4.0, S: Denotes source that is static (e.g. a table from a paper, a data download from a paper, or a source that has been deprecated), U: Denotes source that has been updated in the DGIdb 4.0. *ChEMBL is listed twice as one source (interactions) is aggregated by DGIdb, while the other (drugs) is part of the normalizer used by DGIdb.

Supplementary Table 2: Interaction types, definitions, and directionalities

**Interaction Type**	**Definition**	**Directionality**
activator	An activator interaction is when a drug activates a biological response from a target, although the mechanism by which it does so may not be understood.	activating
adduct	An adduct interaction is when a drug-protein adduct forms by the covalent binding of electrophilic drugs or their reactive metabolite(s) to a target protein.	N/A
agonist	An agonist interaction occurs when a drug binds to a target receptor and activates the receptor to produce a biological response.	activating
allosteric modulator	An allosteric modulator interaction occurs when drugs exert their effects on their protein targets via a different binding site than the natural (orthosteric) ligand site.	N/A
antagonist	An antagonist interaction occurs when a drug blocks or dampens agonist-mediated responses rather than provoking a biological response itself upon binding to a target receptor.	inhibitory

antibody	An antibody interaction occurs when an antibody drug specifically binds the target molecule.	inhibitory
antisense oligonucleotide	An antisense oligonucleotide interaction occurs when a complementary RNA drug binds to an mRNA target to inhibit translation by physically obstructing the mRNA translation machinery.	inhibitory
binder	A binder interaction has drugs physically binding to their target.	N/A
blocker	Antagonist interactions are sometimes referred to as blocker interactions; examples include alpha blockers, beta blockers, and calcium channel blockers.	inhibitory
chaperone	Pharmacological chaperone interactions occur when substrates or modulators directly bind to a partially folded biosynthetic intermediate to stabilise the protein and allow it to complete the folding process to yield a functional protein.	activating
cleavage	Cleavage interactions take place when the drug promotes degeneration of the target protein through cleaving of the peptide bonds.	inhibitory
cofactor	A cofactor is a drug that is required for a target protein's biological activity.	activating
inducer	In inducer interactions, the drug increases the activity of its target enzyme.	activating
inhibitor	In inhibitor interactions, the drug binds to a target and decreases its expression or activity. Most interactions of this class are enzyme inhibitors, which bind an enzyme to reduce enzyme activity.	inhibitory
inhibitory allosteric modulator	In inhibitory allosteric modulator interactions, also called negative allosteric modulator interactions, the drug will inhibit activity of its target enzyme.	inhibitory
inverse agonist	An inverse agonist interaction occurs when a drug binds to the same target as an agonist, but induces a pharmacological response opposite to that of the agonist.	inhibitory
ligand	In ligand interactions, a drug forms a complex with its target protein to serve a biological function.	N/A
modulator	In modulator interactions, the drug regulates or changes the activity of its target. In contrast to allosteric modulators, this interaction type may not involve any direct binding to the target.	N/A
multitarget	In multitarget interactions, drugs achieve a physiological effect through simultaneous interaction with multiple gene targets.	N/A
N/A	DGIdb assigns this label to any drug-gene interaction for which the interaction type is not specified by the reporting source.	N/A
negative modulator	In a negative modulator interaction, the drug negatively regulates the amount or activity of its target. In contrast to an inhibitory allosteric modulator, this interaction type may not involve any direct binding to the target.	inhibitory
other/unknown	This is a label given by the reporting source to an interaction that doesn't belong to other interaction types, as defined by the reporting source.	N/A
partial agonist	In a partial agonist interaction, a drug will elicit a reduced amplitude functional response at its target receptor, as compared to the response elicited by a full	activating

	agonist.	
partial antagonist	In a partial antagonist interaction, a drug will only partially reduce the amplitude of a functional response at its target receptor, as compared to the reduction of response by a full antagonist.	inhibitory
positive modulator	In a positive modulator interaction, the drug increases activity of the target enzyme.	activating
potentiator	In a potentiator interaction, the drug enhances the sensitivity of the target to the target's ligands.	N/A
product of	These "interactions" occur when the target gene produces the endogenous drug.	N/A
stimulator	In a stimulator interaction, the drug directly or indirectly affects its target, stimulating a physiological response.	activating
substrate	Substrates are drugs which are metabolized by the protein they are interacting with.	N/A
suppressor	In a suppressor interaction, the drug directly or indirectly affects its target, suppressing a physiological process.	inhibitory
vaccine	In vaccine interactions, the drugs stimulate or restore an immune response to their target.	activating

FIGURES:

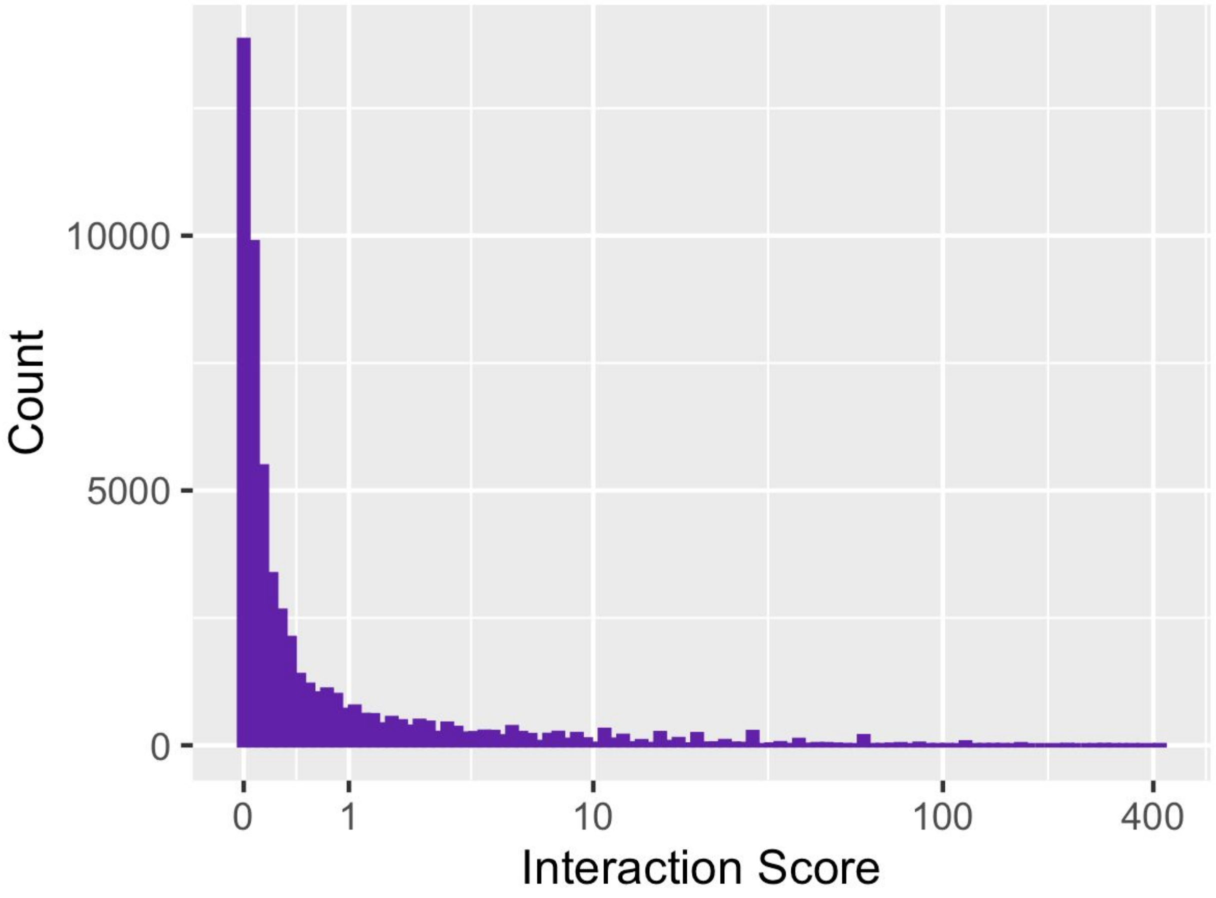

Supplementary Figure 1
Distribution of *Interaction Scores* for all 54,591 drug-gene interaction records currently in DGIdb, scores range from 0.00 to 416.57. Lower scores correspond to interactions that have low Evidence Scores (i.e. are few sources or publications supporting the interaction record) and/or interactions that have promiscuous drugs or genes (meaning the drug, gene, or both the drug and gene involved in the interaction record interact with many other genes or drugs across all records). For example, several of the top 10 most commonly occurring drugs involved in interactions with extremely low *Interaction Scores* (interactions in the first bin of scores) are kinase inhibitors such as *Fostamatinib* and *Cenisertib*. Many of the interactions for these two drugs are reported by only DrugBank or Drug Target Commons (leading to low Evidence Scores of only 1 or 2) and both drugs are highly promiscuous (interacting with 298 and 116 genes, respectively). The average number of known gene partners per drug concept is 4.36. In contrast, higher scores correspond to interactions that have high Evidence Scores and/or low drug or gene promiscuity. For example, two of the top scoring interactions reported are *Elotuzumab*-SLAMF7 and *Nitisinone*-HPD, which are reported by multiple sources (leading to higher Evidence Scores) and have low promiscuity for both drugs and genes. The interaction between *Elotuzumab* and SLAMF7 is the only reported interaction involving either *Elotuzumab*

or SLAMF7. *Nitisinone* is only reported to interact with HPD and HPD only interacts with one additional drug. The average number of known drug partners per gene is 13.0.

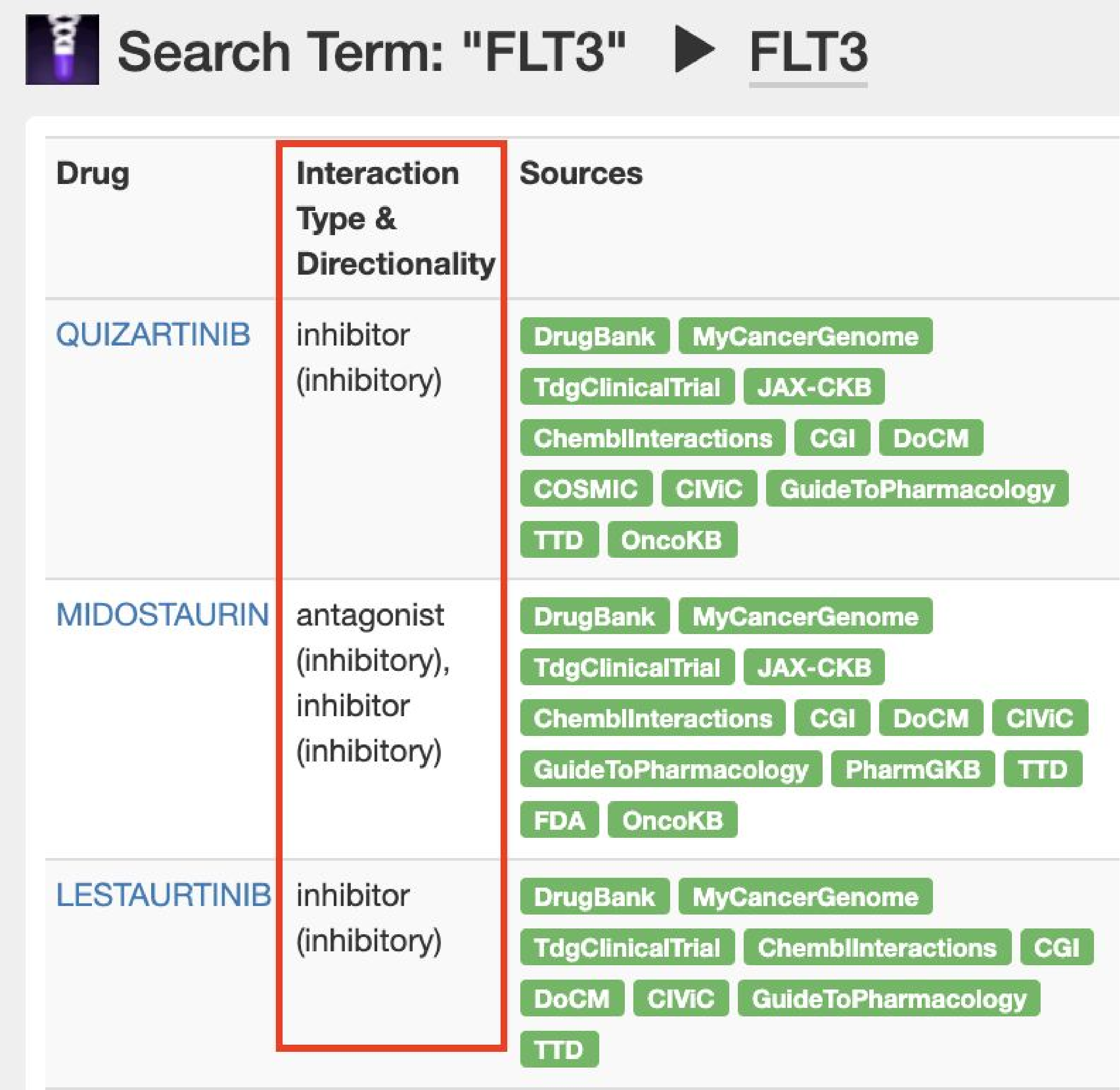

Search Term: "FLT3" ▶ FLT3

Drug	Interaction Type & Directionality	Sources
QUIZARTINIB	inhibitor (inhibitory)	DrugBank, MyCancerGenome, TdgClinicalTrial, JAX-CKB, ChemblInteractions, CGI, DoCM, COSMIC, CIViC, GuideToPharmacology, TTD, OncoKB
MIDOSTAURIN	antagonist (inhibitory), inhibitor (inhibitory)	DrugBank, MyCancerGenome, TdgClinicalTrial, JAX-CKB, ChemblInteractions, CGI, DoCM, CIViC, GuideToPharmacology, PharmGKB, TTD, FDA, OncoKB
LESTAURTINIB	inhibitor (inhibitory)	DrugBank, MyCancerGenome, TdgClinicalTrial, ChemblInteractions, CGI, DoCM, CIViC, GuideToPharmacology, TTD

Supplementary Figure 2
A subset of interacting drugs for the *FLT3* gene. *Interaction type* and *directionality* annotations are outlined in red. The new directionality indication (*inhibitory*) is provided in parentheses after the interaction type (*inhibitor*, *antagonist*) in the interaction search results.

ADDITIONAL Supplementary MATERIAL:

Pseudocode for the DGIdb drug grouper

main

1. Look for ChEMBL normalizer with match >= 80
    a. If match has only one record:
        i. return record normalized to chembl_id
    b. If match has multiple records all with same chembl ID:
        i. return record normalized to chembl_id
    c. If match has multiple records with different chembl IDs:
        i. select record with highest max phase
            1. if multiple records, narrow down to those with a trade name
                a. if still multiple records, use the one with the lowest chembl id and normalized to chembl_id
                b. if one record, normalized to chembl_id
2. Select normalizer(s) with highest match and match > 0:
    a. Further select on normalizers have records with chembl ids, if any
        i. if no normalizers have records with chembl ids, keep all normalizers
    b. Select results from normalizer with highest priority
        i. If selected normalizer is ChEMBL, go to Step 1a
        ii. If match has only one record:
            1. If match has a chembl id and id is valid
                a. return record normalized to chembl ID
            2. Else
                a. return record
        iii. if match has multiple records:
            1. select any records with a chembl id that is valid
                a. if the chembl ids are the same, return record normalized to chembl_id
                b. if the chembl ids are not the same:
                    i. normalize each record to chembl ID
                    ii. if only one record has highest max_phase:
                        1. return normalized record with greatest max_phase
                    iii. else:
                        1. if match score >= 40:
                            a. narrow down to those with a trade name
                                i. if still multiple records, use the one with the lowest chembl id and normalized to chembl_id
                                ii. if one record, normalized to chembl_id
                        2. if match score < 40:
                            a. do not normalize
            2. if no records have a chembl id
                a. do not normalize
3. If no normalizer(s) with match > 0:
    a. do not normalize

records with chembl ids

a record has a chembl ID as the concept_identifier from the chembl normalizer, or may contain one as a CURIE in the other_identifiers field namespaced with “chembl:”

normalize record to chembl ID

NOTE: it may be beneficial to cache this operation on a given chembl ID, as the result will not change.

1. run normalizer query on chembl ID

2. select all normalizers with match >= 80
    a. if a chembl record exists:
        i. create copy of chembl record to *new_record*
        ii. add *aliases* and *other_identifiers* from other normalizers to *new_record*
        iii. return *new_record*
    b. if a chembl record doesn't exist
        i. return None

priority ranking
1. ChEMBL normalizer
2. Wikidata normalizer

References

1. Edwards,A.M., Isserlin,R., Bader,G.D., Frye,S.V., Willson,T.M. and Yu,F.H. (2011) Too many roads not taken. *Nature*, **470**, 163–165.

2. Tamborero,D., Rubio-Perez,C., Deu-Pons,J., Schroeder,M.P., Vivancos,A., Rovira,A. and Others Cancer Genome Interpreter annotates the biological and clinical relevance of tumor alterations. Genome Med. 2018; 10 (1): 25 Epub 2018/03/30. https://doi. org/10.1186/s13073-018-0531-8 PMID: 29592813.

3. Shrager,J., Tenenbaum,J.M. and Travers,M. (2011) Cancer Commons: Biomedicine in the Internet Age. In Ekins,S., Hupcey,M.A.Z., Williams,A.J. (eds), *Collaborative Computational Technologies for Biomedical Research*, Biostatistics Series. John Wiley & Sons, Inc., Hoboken, NJ, USA, Vol. 465, pp. 161–177.

4. Mendez,D., Gaulton,A., Bento,A.P., Chambers,J., De Veij,M., Félix,E., Magariños,M.P., Mosquera,J.F., Mutowo,P., Nowotka,M., *et al.* (2019) ChEMBL: towards direct deposition of bioassay data. *Nucleic Acids Res.*, **47**, D930–D940.

5. Griffith,M., Spies,N.C., Krysiak,K., McMichael,J.F., Coffman,A.C., Danos,A.M., Ainscough,B.J., Ramirez,C.A., Rieke,D.T., Kujan,L., *et al.* (2017) CIViC is a community knowledgebase for expert crowdsourcing the clinical interpretation of variants in cancer. *Nat. Genet.*, **49**, 170–174.

6. Tate,J.G., Bamford,S., Jubb,H.C., Sondka,Z., Beare,D.M., Bindal,N., Boutselakis,H., Cole,C.G., Creatore,C., Dawson,E., *et al.* (2019) COSMIC: the Catalogue Of Somatic Mutations In Cancer. *Nucleic Acids Res.*, **47**, D941–D947.

7. Kumar,R.D., Chang,L.-W., Ellis,M.J. and Bose,R. (2013) Prioritizing Potentially Druggable Mutations with dGene: An Annotation Tool for Cancer Genome Sequencing Data. *PLoS One*, **8**, e67980.

8. Ainscough,B.J., Griffith,M., Coffman,A.C., Wagner,A.H., Kunisaki,J., Choudhary,M.N., McMichael,J.F., Fulton,R.S., Wilson,R.K., Griffith,O.L., *et al.* (2016) DoCM: a database of curated mutations in cancer. *Nat. Methods*, **13**, 806–807.

9. Tanoli,Z., Alam,Z., Vähä-Koskela,M., Ravikumar,B., Malyutina,A., Jaiswal,A., Tang,J., Wennerberg,K. and Aittokallio,T. (2018) Drug Target Commons 2.0: a community platform

for systematic analysis of drug-target interaction profiles. *Database* , **2018**, 1–13.

10. Wishart,D.S., Feunang,Y.D., Guo,A.C., Lo,E.J., Marcu,A., Grant,J.R., Sajed,T., Johnson,D., Li,C., Sayeeda,Z., *et al.* (2018) DrugBank 5.0: a major update to the DrugBank database for 2018. *Nucleic Acids Res.*, **46**, D1074–D1082.

11. Yates,A.D., Achuthan,P., Akanni,W., Allen,J., Allen,J., Alvarez-Jarreta,J., Amode,M.R., Armean,I.M., Azov,A.G., Bennett,R., *et al.* (2020) Ensembl 2020. *Nucleic Acids Res.*, **48**, D682–D688.

12. Wagle,N., Berger,M.F., Davis,M.J., Blumenstiel,B., Defelice,M., Pochanard,P., Ducar,M., Van Hummelen,P., Macconaill,L.E., Hahn,W.C., *et al.* (2012) High-throughput detection of actionable genomic alterations in clinical tumor samples by targeted, massively parallel sequencing. *Cancer Discov.*, **2**, 82–93.

13. Ashburner,M., Ball,C.A., Blake,J.A., Botstein,D., Butler,H., Cherry,J.M., Davis,A.P., Dolinski,K., Dwight,S.S., Eppig,J.T., *et al.* (2000) Gene ontology: tool for the unification of biology. The Gene Ontology Consortium. *Nat. Genet.*, **25**, 25–29.

14. The Gene Ontology Consortium (2019) The Gene Ontology Resource: 20 years and still GOing strong. *Nucleic Acids Res.*, **47**, D330–D338.

15. Armstrong,J.F., Faccenda,E., Harding,S.D., Pawson,A.J., Southan,C., Sharman,J.L., Campo,B., Cavanagh,D.R., Alexander,S.P.H., Davenport,A.P., *et al.* (2020) The IUPHAR/BPS Guide to PHARMACOLOGY in 2020: extending immunopharmacology content and introducing the IUPHAR/MMV Guide to MALARIA PHARMACOLOGY. *Nucleic Acids Res.*, **48**, D1006–D1021.

16. Finan,C., Gaulton,A., Kruger,F.A., Lumbers,R.T., Shah,T., Engmann,J., Galver,L., Kelley,R., Karlsson,A., Santos,R., *et al.* (2017) The druggable genome and support for target identification and validation in drug development. *Sci. Transl. Med.*, **9**.

17. Hopkins,A.L. and Groom,C.R. (2002) The druggable genome. *Nat. Rev. Drug Discov.*, **1**, 727–730.

18. Uhlén,M., Fagerberg,L., Hallström,B.M., Lindskog,C., Oksvold,P., Mardinoglu,A., Sivertsson,Å., Kampf,C., Sjöstedt,E., Asplund,A., *et al.* (2015) Proteomics. Tissue-based map of the human proteome. *Science*, **347**, 1260419.

19. Rodgers,G., Austin,C., Anderson,J., Pawlyk,A., Colvis,C., Margolis,R. and Baker,J. (2018) Glimmers in illuminating the druggable genome. *Nat. Rev. Drug Discov.*, **17**, 301–302.

20. Patterson,S.E., Liu,R., Statz,C.M., Durkin,D., Lakshminarayana,A. and Mockus,S.M. (2016) The clinical trial landscape in oncology and connectivity of somatic mutational profiles to targeted therapies. *Hum. Genomics*, **10**, 4.

21. Cheng,D.T., Mitchell,T.N., Zehir,A., Shah,R.H., Benayed,R., Syed,A., Chandramohan,R., Liu,Z.Y., Won,H.H., Scott,S.N., *et al.* (2015) Memorial Sloan Kettering-Integrated Mutation Profiling of Actionable Cancer Targets (MSK-IMPACT): A Hybridization Capture-Based Next-Generation Sequencing Clinical Assay for Solid Tumor Molecular Oncology. *J. Mol.*

Diagn., **17**, 251–264.

22. Jain,N., Mittendorf,K.F., Holt,M., Lenoue-Newton,M., Maurer,I., Miller,C., Stachowiak,M., Botyrius,M., Cole,J., Micheel,C., *et al.* (2020) The My Cancer Genome clinical trial data model and trial curation workflow. *J. Am. Med. Inform. Assoc.*, **27**, 1057–1066.

23. Brown,G.R., Hem,V., Katz,K.S., Ovetsky,M., Wallin,C., Ermolaeva,O., Tolstoy,I., Tatusova,T., Pruitt,K.D., Maglott,D.R., *et al.* (2015) Gene: a gene-centered information resource at NCBI. *Nucleic Acids Res.*, **43**, D36–42.

24. Chakravarty,D., Gao,J., Phillips,S.M., Kundra,R., Zhang,H., Wang,J., Rudolph,J.E., Yaeger,R., Soumerai,T., Nissan,M.H., *et al.* (2017) OncoKB: A Precision Oncology Knowledge Base. *JCO Precis Oncol*, **2017**.

25. Williams,H.L., Walsh,K., Diamond,A., Oniscu,A. and Deans,Z.C. (2018) Validation of the Oncomine^{TM} focus panel for next-generation sequencing of clinical tumour samples. *Virchows Arch.*, **473**, 489–503.

26. Whirl-Carrillo,M., McDonagh,E.M., Hebert,J.M., Gong,L., Sangkuhl,K., Thorn,C.F., Altman,R.B. and Klein,T.E. (2012) Pharmacogenomics knowledge for personalized medicine. *Clin. Pharmacol. Ther.*, **92**, 414–417.

27. Nguyen,D.-T., Mathias,S., Bologa,C., Brunak,S., Fernandez,N., Gaulton,A., Hersey,A., Holmes,J., Jensen,L.J., Karlsson,A., *et al.* (2017) Pharos: Collating protein information to shed light on the druggable genome. *Nucleic Acids Res.*, **45**, D995–D1002.

28. Kim,S., Chen,J., Cheng,T., Gindulyte,A., He,J., He,S., Li,Q., Shoemaker,B.A., Thiessen,P.A., Yu,B., *et al.* (2019) PubChem 2019 update: improved access to chemical data. *Nucleic Acids Res.*, **47**, D1102–D1109.

29. Russ,A.P. and Lampel,S. (2005) The druggable genome: an update. *Drug Discov. Today*, **10**, 1607–1610.

30. Morgensztern,D., Campo,M.J., Dahlberg,S.E., Doebele,R.C., Garon,E., Gerber,D.E., Goldberg,S.B., Hammerman,P.S., Heist,R.S., Hensing,T., *et al.* (2015) Molecularly targeted therapies in non-small-cell lung cancer annual update 2014. *J. Thorac. Oncol.*, **10**, S1–63.

31. Rask-Andersen,M., Masuram,S. and Schiöth,H.B. (2014) The druggable genome: Evaluation of drug targets in clinical trials suggests major shifts in molecular class and indication. *Annu. Rev. Pharmacol. Toxicol.*, **54**, 9–26.

32. Beaubier,N., Tell,R., Lau,D., Parsons,J.R., Bush,S., Perera,J., Sorrells,S., Baker,T., Chang,A., Michuda,J., *et al.* (2019) Clinical validation of the tempus xT next-generation targeted oncology sequencing assay. *Oncotarget*, **10**, 2384–2396.

33. Wang,Y., Zhang,S., Li,F., Zhou,Y., Zhang,Y., Wang,Z., Zhang,R., Zhu,J., Ren,Y., Tan,Y., *et al.* (2020) Therapeutic target database 2020: enriched resource for facilitating research and early development of targeted therapeutics. *Nucleic Acids Res.*, **48**, D1031–D1041.

34. Rask-Andersen,M., Almén,M.S. and Schiöth,H.B. (2011) Trends in the exploitation of novel

drug targets. *Nat. Rev. Drug Discov.*, **10**, 579–590.

35. Vrandečić,D. and Krötzsch,M. (2014) Wikidata: a free collaborative knowledgebase. *Commun. ACM*, **57**, 78–85.
